# Multiscale conformal pattern transfer

**DOI:** 10.1038/srep28490

**Published:** 2016-06-22

**Authors:** Kristof Lodewijks, Vladimir Miljkovic, Inès Massiot, Addis Mekonnen, Ruggero Verre, Eva Olsson, Alexandre Dmitriev

**Affiliations:** 1 Department of Physics, Chalmers University of Technology, 41296 Gothenburg, Sweden; 2 Department of Physics, University of Gothenburg, 41296 Gothenburg, Sweden

## Abstract

We demonstrate a method for seamless transfer from a parent flat substrate of basically any lithographic top-down or bottom-up pattern onto essentially any kind of surface. The nano- or microscale patterns, spanning macroscopic surface areas, can be transferred with high conformity onto a large variety of surfaces when such patterns are produced on a thin carbon film, grown on top of a sacrificial layer. The latter allows lifting the patterns from the flat parent substrate onto a water-air interface to be picked up by the host surface of choice. We illustrate the power of this technique by functionalizing broad range of materials including glass, plastics, metals, rough semiconductors and polymers, highlighting the potential applications in *in situ* colorimetry of the chemistry of materials, anti-counterfeit technologies, biomolecular and biomedical studies, light-matter interactions at the nanoscale, conformal photovoltaics and flexible electronics.

The rapid development of micro- and nanofabrication produced a true technological revolution. This fast progress is possible by the development of various lithography techniques that are driven by the strong demand of scaling down integrated circuits. While modern superior chip-based fabrication has reached the few-nanometers scale, its applicability beyond the context of advanced electronics, for example, in functionalizing soft, polymeric or organic materials or materials with large natural roughness, is hindered by the essential need of a well-defined flat substrate as a support. In recent years, advanced optical lithography techniques such as immersion lithography^1^, multiple exposure steps and the usage of extreme ultraviolet (EUV) light sources^2^ allowed to scale down the smallest possible feature sizes down to about 10 nanometers, while achieving defect-free, registered, arbitrary patterns on wafer-scale surfaces. Similar pattern resolution is reached with sequential lithography techniques such as electron beam lithography (EBL)^3^, focused ion beam and direct laser writing^4^, albeit for much smaller surface areas due to the limited throughput of these techniques. The emergence of nanoimprint lithography^5^ and soft-lithography^6^ allowed to increase the throughput of these lab-based methods substantially by multiple usage of the same patterned stamps. Next to the conventional top-down technologies, many bottom-up approaches have been developed recently, allowing to pattern large areas with periodic or aperiodic micro- and nanoscale features. Among a large number of low-cost fabrication techniques^7^, colloidal lithography^8^,^9^ is probably the most known. Many more advanced patterning technologies have emerged, such as self-assembled monolayers (SAMs)^10^, DNA- templating^11^, atomic and molecular engineering^12^ and functional block copolymers^13^ to name a few. However, the fact that an overwhelming number of patterning methods mainly function on 2D and often atomically flat surfaces puts severe limitations onto their usability in a broad range of every-day-use or advanced technologies, where materials with non-planar, soft, mechanically agile or chemically sensitive surfaces or surfaces with large natural roughness are used. To that extent, in recent years, several transfer protocols have been proposed to overcome this limitation^14^,^15^,^16^,^17^,^18^,^19^,^20^.

Here we developed a particularly versatile protocol, allowing the transfer of essentially any lithographically defined micro- or nanostructured top-down or bottom- up pattern from a flat parent substrate onto a randomly shaped three-dimensional host. The transferred pattern efficiently conforms to the shape of the host from nano- to micro- and macroscale, functionalizing the entire surface with a precisely defined nano- or micro-pattern. The key is the use of a 10 nm-thin amorphous carbon film as a pattern-supporting layer that enables highly conformal and easy transfer. As such, a 10 nm carbon film combines extreme strength with a large degree of flexibility, thereby capable of adapting its shape to a three-dimensional host object. The flexibility of our approach is what distinguishes this work from previously reported techniques^14^,^15^,^16^,^17^,^18^,^19^,^20^, as the limited thickness of the carbon film facilitates conformation of the transferred pattern to nearly any macroscopic or microscopic 3D object.

Figure 1 outlines the sequence of steps to achieve the pattern transfer (for more details see the Methods section and Supplementary info, Fig. S1). First, a sacrificial lift- off layer is deposited onto the parent substrate by a deposition method that depends on the choice of the material. In the examples that follow we chose silicon oxide (100 nm of sputter-deposited SiO_2_) for the ease of deposition and removal by a standard buffered oxide etch (BOE or buffered hydrofluoric acid, HF). We note that the choice of a sacrificial layer is open for a large number of materials, as long as their etching conditions are distinctly different from those of the carbon film and the pattern. We then deposit a 10 nm carbon film by e-beam evaporation, followed by 500 °C bake in argon atmosphere to increase the layer strength and stability. The lithographic pattern for transfer is then defined on top of the carbon film by essentially any bottom-up or top- down lithography method at hand. Subsequently, for the pattern transfer, the sacrificial layer is removed by a wet etch, and the parent substrate is used to pick up the carbon film with the pattern from the etch solution. Immersing it in water makes the largely intact hydrophobic carbon film with the pattern to float at the air-water interface, from which it will be picked by the host. Prior to this step, the host substrate is treated with oxygen plasma in order to make it hydrophilic.

The combination of the hydrophilicity of the host and the hydrophobicity of the carbon carbon film eases the drying process by gentle nitrogen flow and reduces the potential damage due to occasional water droplets moving around below the pattern. If required by the functionality of the pattern, for example, if the exact dielectric environment of the pattern is important for certain applications, the carbon film is etched using isotropic oxygen plasma. The possibility to completely remove the carbon film under the structure depends on the scale and mechanical properties of the transferred pattern. In several examples below, the nearly complete removal of the carbon film is illustrated on discrete nanostructures.

With such protocol at hand it turns out we open virtually unlimited opportunities to functionalize the hosts that are utilized in the broadest range of fields of science and technology. In what follows, we test this range by applying the method in contexts that differ in pattern scale by many orders of magnitude, in mechanical and surface characteristics of the host and potential environments the functionalized host would be exposed to. Figures 2 and 3 outline the landscape of these explored opportunities.

For the first set of examples, the transferred pattern consists of a bottom-up short-range ordered pattern of Au nanoplasmonic disk antennas with different diameter and 30 nm height (see electron micrograph (SEM) of the transferred pattern bottom-left of Fig. 2a), with glass microscope slide as a parent substrate, and fabricated by hole-mask colloidal lithography (HCL)^9^. It is easy to follow the different stages in the fabrication process (Fig. 1) colorimetrically by tracking the color of the particle patterns, in this case for a nominal diameter of 100 nm (Fig. 2a). The plasmonic nanoparticles are known to resonantly scatter and absorb visible light, producing easily detectable colors^21^. While the pristine nanoplasmonic array scatters in vivid light turquoise, same array on the carbon film is seen darker with bleaker yet distinct color (Fig. 2a top-left). After the transfer onto another glass microscope slide, the array maintains its darker hue (Fig. 2a, top-right - note the clean carbon film seen dark grey), and recovers the original color after etching away the carbon film (Fig. 2a, bottom- right). The latter signals that the protocol preserves the properties of the transferred pattern to a large extent - something that will be discussed in more detail below.

We stay with this geometrically and spectrally well- defined colorimetric nanoplasmonic system to evaluate the quality of the pattern transfer onto real-life macroscopic objects. First we take a commercial light bulb for its curved thin-glass fragile surface. Figure 2b (left panel) shows the amorphous array of 60-nm- diameter Au nanodisk antennas (thus pink appearance - compare to 100 nm-nanodisks of Fig. 2a), placed on the surface of a bulb, easily conforming to the surface curvature (carbon film etched by oxygen plasma). Same large- scale portions of the nanoplasmonic array can also be transferred onto a material with a markedly different surface texture like paper banknotes (Fig. 2b, right panel, carbon film removed by oxygen plasma). Note that our colorimetric arrays immediately report the drastic change in their dielectric environment, as now we employ 100 nm diameter plasmon nanodisk antennas (as in Fig. 2a). On banknotes they display dark-pink color, sensing the contrast between glass and cellulose. The combination of the sub-diffraction nanoscopic structure of the amorphous array, only discernable with the electron microscope, the visual color with associated optical spectral signature and the possibility to controllably shape the array patch on a macroscale by pre-cutting the carbon film with the pattern prior to transfer (see rectangular shaped transferred array in Fig. 2b, right panel) makes the transfer protocol very suitable for the design of next-generation anti-counterfeit^22^ optical labels for a broad range of consumer products. The intact transferred areas easily reach about 1 cm^2^, which could potentially be improved when using alternative substrate materials with a higher baking temperature to improve the morphology of the carbon film. The annealing step used in these examples indeed increases the strength and stability of the amorphous carbon film, but is not sufficiently high to induce a very high degree of recrystallization. The main limiting factor in the achieved areas is the manual handling of the substrates in the different steps of the transfer, something which can be circumvented with dedicated tools that can ease the transfer, similar to what was shown before^20^. Wrinkling is another potential pitfall that can be circumvented to a large extent by making the host substrate hydrophilic using a short oxygen plasma treatment prior to transfer.

Next we test the pattern transfer as a method for facile addition of the nano-functionality to the biomolecular technology. We start with a standard polydimethylsiloxane (PDMS) microfluidic chip with 3 μm*-*deep channels, commonly employed in biomolecular studies^23^, and transfer an array of 100 nm-diameter plasmon nanodisk antennas onto the chip (for more examples see Fig. S3, Supplementary information). In Fig. 3c (left) the overall geometry of the channel with inlet area is visible, covered with turquoise-colored plasmonic array (compare the array color with Fig. 2a, bottom- right - PDMS has refractive index close to glass). High- resolution SEM (Fig. 2c, right, and zoomed-in area of the channel wall) displays the unperturbed nanoplasmonic pattern, uniformly decorating the sidewalls and the bottom of the channel, even after removal of the carbon film. For certain applications, it might be required to increase the adhesion between the transferred structures and the host substrate, which can be achieved by annealing the structures before etching the carbon film, or by specific chemical functionalization of the host. Such facile functionalization potentially gives room to a large variety of microfluidics-based biomolecular studies at a nanoscale, including sensing, enhancement of photo-induced processes with light- capturing plasmon nanoantennas or, when varying the material of the nanopattern, in bio-nanocatalysis and energy storage^24^. The conformity of the pattern to the host surface can be visualized also by functionalizing a close-packed layer of 2 μm-sized polystyrene beads (Fig. 2d). The SEM reveals a nanoplasmonic ‘carpet’ of 150 nm-diameter Au antennas, decorating the bead array. Due to the features density of the host, the Au antennas are ‘hanging’ in-between the supporting bead pattern. We foresee more biomolecular scenarios where micro- and nanoscale decoration and conformity would play a central role. For example, we anticipate the possible decoration of surface-confined large and small biomolecules with an overlaying carbon film and light- capturing nanoantennas to produce hybrid systems for enhanced photochemistry. Another example can be the use of carefully optimized nano-geometries for surface- enhanced Raman spectroscopy that can be wrapped around microscopic or macroscopic probed objects^25^. In this example, we did not etch the carbon layer, but one could expect the eventual amorphous arrays of nanoparticles with local density gradients when both the carbon film and beads are etched with oxygen plasma.

The structural properties of the nanostructures can be explored with atomic resolution using the pattern transfer. For this we transfer the amorphous array of 100 nm-diameter Au antennas onto conventional transmission electron microscope (TEM) grid (Fig. 2e, left panel - SEM of one of the windows with a nanodisk pattern, inset - a photo of the carbon film on the grid). Panels on the right of Fig. 2e display a successively zoomed-in TEMs of the nanodisks arrangement, single nanodisk antenna, satellite particles on the side of the nanodisk and, finally, atomically- resolved approximately 2 nm-sized nanoparticle. The satellite particles surrounding the nanodisks are inherent to the HCL fabrication process, as during the evaporation of the particles, atoms scatter from the edges of the hole mask and agglomerate in small islands surrounding the main particle. With the notion of TEM as a superior instrument for structural investigations at atomic scale, but also a tool for the rapidly growing studies of light-matter interactions at the nanoscale^26^,^27^, the proposed transfer with arbitrary pattern design opens exciting possibilities.

Up to now we have tested the transfer of patterns of nanostructures. Figure 3a instead presents a continuous nano-perforated thin platinum film transferred onto a polycrystalline silicon substrate. Polycrystalline silicon (poly-Si) has been developed on industrial scale as a cheap alternative to high-quality crystalline silicon for solar cells, with polycrystalline silicon modules reaching similar efficiencies as monocrystalline silicon ones^28^. Its natural roughness (see Fig. 2a) presents a significant challenge for further functionalization aimed at improving the efficiency of poly-Si photovoltaic devices. Here the pattern transfer allows continuous (up to 50 μm) areas of metallic film (perforated with 170 nm-nanoholes for enhanced light-trapping in the near-infrared range) and intended as a front electrode to be conformed to the poly-Si surface with microscopic roughness (see also Fig. S4, Supporting information). We expect that the transfer of patterned or pristine thin films of various materials should be permitted with the same protocol, opening the route towards conformal thin-film photovoltaics or light generation and detection technologies.

In the last example the transfer of a more intricate electron-beam-lithography fabricated micro-pattern is shown. Figure 3b demonstrates the logo of Chalmers University of Technology, transferred onto the top surface of a glass consumer-grade white light-emitting diode (LED) (Fig. 3b, left panels). The quality of the transferred pattern is confirmed by directing the light from LED to a simple microscope-optics-based projection setup (Fig. 3b, right panel). Here the sub-100 μm logo on the LED surface acts as a projection mask, with its several-cm-sized image (that is, with 500× effective magnification), projected onto a lab wall. Lower magnification microscope views and SEMs before and after transfer can be found in Supplementary information (Fig. S5). With the micropattern transfer, possibilities are opening to realize in practice new conformal metadevices^24^ that could perform their intended optical function (like electromagnetic cloaking^29^) on arbitrarily shaped objects, but also possibly in stretchable micro-electronics, where electronic component functionalities usually depend on their fabrication directly on elastomeric (PDMS) membranes^30^. With the latter, it is very challenging to reliably achieve the multiple patterning steps, so the pattern transfer could potentially allow fully defining the desired electronic configuration on a parent substrate and then transferring it onto an elastomer, similarly to how it is done on PDMS in the present study (Fig. 2c).

Finally, we use the plasmonic properties of nanoparticle arrays to spectroscopically follow different steps of the transfer protocol. For this we use Au nanodisk antennas of various diameters (Fig. 4a), producing different optical spectra and visual color (inset of Fig. 4a). Similarly to Fig. 2a, here the fabrication on a carbon film (Fig. 4b) results in color change, spectroscopically detected as the plasmon resonance red shift due to a higher refractive index environment and with resonance broadening due to additional light absorption in the carbon film. Etching away the latter restores the color and resonances positions (Fig. 4c). The minor blue shift (with respect to the reference samples in Fig. 4a) can be possibly attributed to a small reduction in size of the gold nanodisks due to the oxygen plasma etch. Further, reproducing the transfer onto commercial-grade plastic white-light LEDs (similar to Fig. 3b), we are able to test the direct transfer of nanoplasmonic arrays onto the LED surface, with the visible spectral change of the array (Fig. 4d), namely resonance blue shift due to the lower refractive index of the plastic and resonance broadening that can be attributed to pattern distortion due to the large roughness. These spectral changes are also reflected in the change of the LED light colors (see the inset of Fig. 3d). Detailed visualizations of the colorimetric response are found in Supplementary information in Fig. S2 (nanodisks on glass) and in Fig. S6 (transfer onto LEDs), along with the photos at different steps of the process.

In summary, we developed a simple, affordable and a versatile protocol to transfer bottom-up or top- down nano- or microscale lithographic patterns of choice onto essentially any host surface. We demonstrate how various lithographic patterns, defined at various length scales, can be adopted with high conformity to functionalize a broad range of surfaces and materials including glass, plastics, metals, rough semiconductors and polymers. The contexts of potential applications of this technique include, but not limited to, *in situ* colorimetry of the chemistry of the materials, anti- counterfeit technologies, biomolecular and biomedical studies, light-matter interactions at the nanoscale, conformal photovoltaics and flexible electronics. The simple protocol spans applications in nano- and microtechnology, essentially linking the intricate nanofabrication to the possible consumer and research applications.

## Experimental Section

### Transfer protocol

The flat parent substrates are cleaned with a conventional cleaning protocol, depending on the substrate material used. The sacrificial lift-off layer (typical thickness 100 nm) is deposited with any conventional deposition technique, depending on the material of choice. A 10 nm carbon film is deposited by e-beam evaporation, followed by 500 °C bake in argon atmosphere to increase the layer strength and stability. The pattern to be transferred is then defined by a conventional lithography technique of choice. For the transfer step, the parent substrate is submerged in a wet etching solution which is selective to the sacrificial layer, but does not affect the pattern to be transferred and the carbon film. Subsequently, the parent substrate is used to pick up the carbon film with the pattern in order to transfer it to the air/water interface. Due to the hydrophobic nature of carbon, the film will float on top of the water solution, exposing the pattern to be transferred to the air. The arbitrarily shaped host substrate is treated with a short oxygen plasma, in order to make it hydrophilic, enabling the conformal pattern transfer. The carbon film and the pattern are then picked up by the host substrate and dried under a gentle nitrogen flow. Depending on the application, the carbon film can be removed in the final step, using an isotropic oxygen plasma etch.

### Alternative material combinations

Depending on the desired application, the material choices can be adjusted in order to transfer a wide variety of nano- and microstructures. The chemical inertness of carbon to most wet etching solutions opens up nearly unlimited options for material combinations for the pattern and the lift-off layer^31^,^32^. For the former, the chemical inertness of the pattern to the etching solution is the only limiting factor, while for the latter more constraints do apply: (i) The lift-off layer must withstand the high temperature baking step used to strengthen the carbon film. Suitable candidates include - but are not limited to - oxides, nitrides and metals with high melting points. (ii) The structures to be transferred should not be affected by oxygen plasma etching, unless an alternative technique is used to remove the carbon film after transfer, or if the removal is unnecessary.

## Additional Information

**How to cite this article**: Lodewijks, K. *et al*. Multiscale conformal pattern transfer. *Sci. Rep.* **6**, 28490; doi: 10.1038/srep28490 (2016).

## Supplementary Material

Supplementary Information

## Figures and Tables

**Figure 1:**
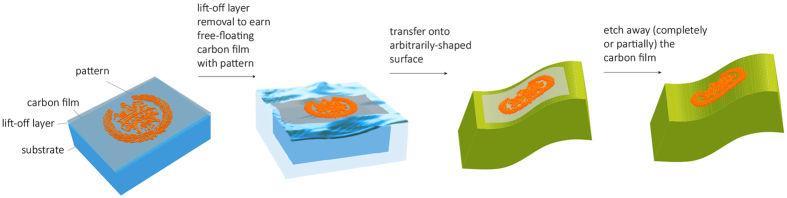
Schematic overview of the pattern transfer. A lithographic pattern is defined on the parent substrate on top of a lift-off sacrificial layer and a carbon film (step 1, far left). After wet etching the lift-off layer, the parent substrate is placed in water to allow the carbon film transfer to the air-water interface due to the natural hydrophobicity of carbon (step 2). The free-floating carbon film is picked up by the arbitrarily shaped host and is then dried under nitrogen flow (step 3). If required, the carbon film is completely or partially removed using an isotropic oxygen plasma etch (step 4).

**Figure 2:**
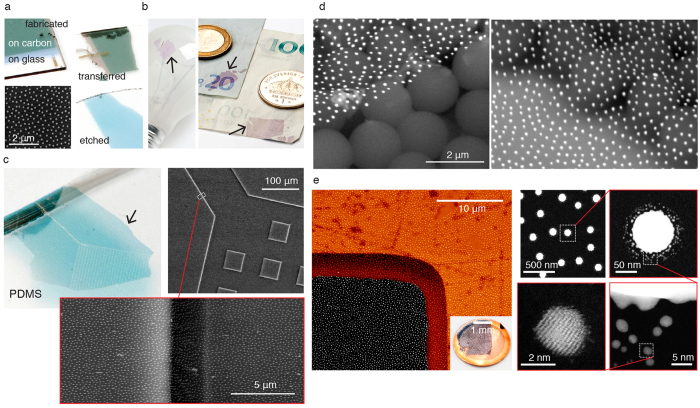
Nanostructures pattern transfer. (**a**) Bottom-up amorphous array of 100 nm-diameter plasmon nanodisk antennas (SEM, bottom-left) on the parent glass slide and carbon film (top-left), transferred to a host glass slide (top-right) and with carbon film removed by etching (bottom-right); (**b**) 60 nm-diameter plasmonic nanodisks, transferred onto a conventional light bulb (left) and 100 nm-diameter nanodisks on banknotes (right); (**c**) 100 nm nanodisk antennas array on microfluidic chip with 3 μm channel depth (photograph of the chip - left, SEM of the inlet channel with zoomed-in side wall - right); (**d**) 150 nm nanodisks decorating the close-packed arrangement of 2 μm polystyrene beads (carbon film not removed, transparent in SEM); (**e**) 100 nm nanodisks array in TEM (left - SEM of the corner of the grid and a photograph of the entire sample, right - successively zoomed-in TEM images of the nanodisks in the array).

**Figure 3:**
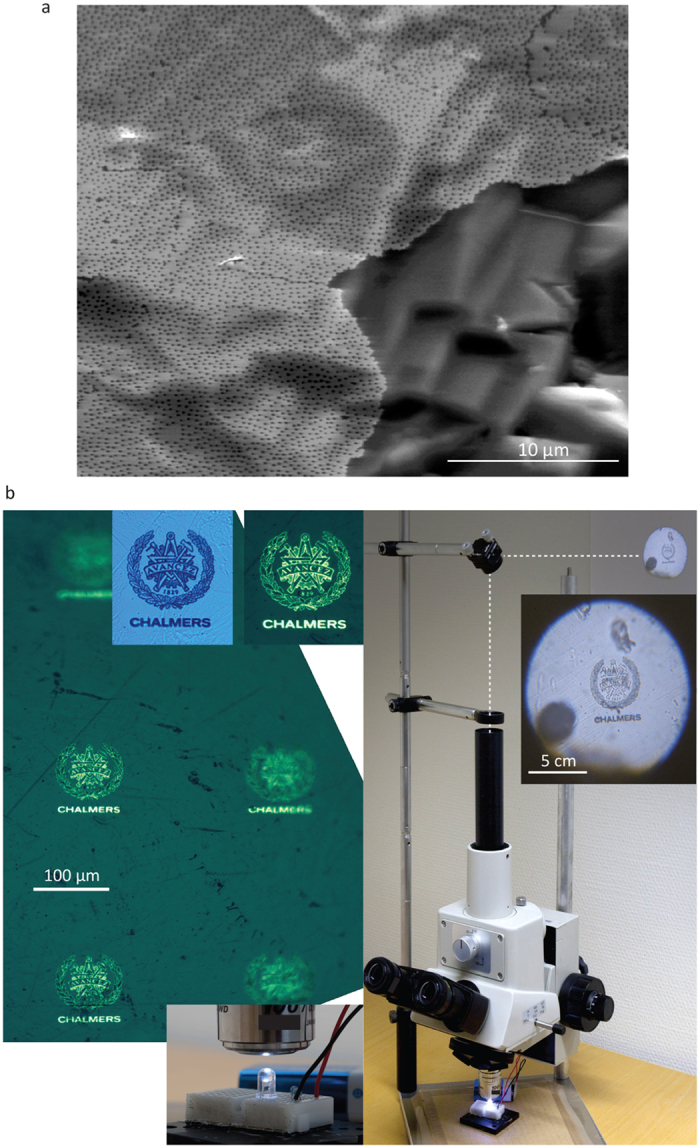
Transfer of nanopatterned thin films and lithographic microstructures. (**a**) Platinum thin film with patterned 150 nm holes on a rough polycrystalline silicon; (**b**) e-beam patterned logo of Chalmers, transferred onto commercial low-cost white-light LED with plastic cover. Microscope images of the decorated LED surface (left, insets - logo imaged using the LED light (left) and microscope light (right)). Microscope- based projection setup to display the LED-logo on the lab wall.

**Figure 4:**
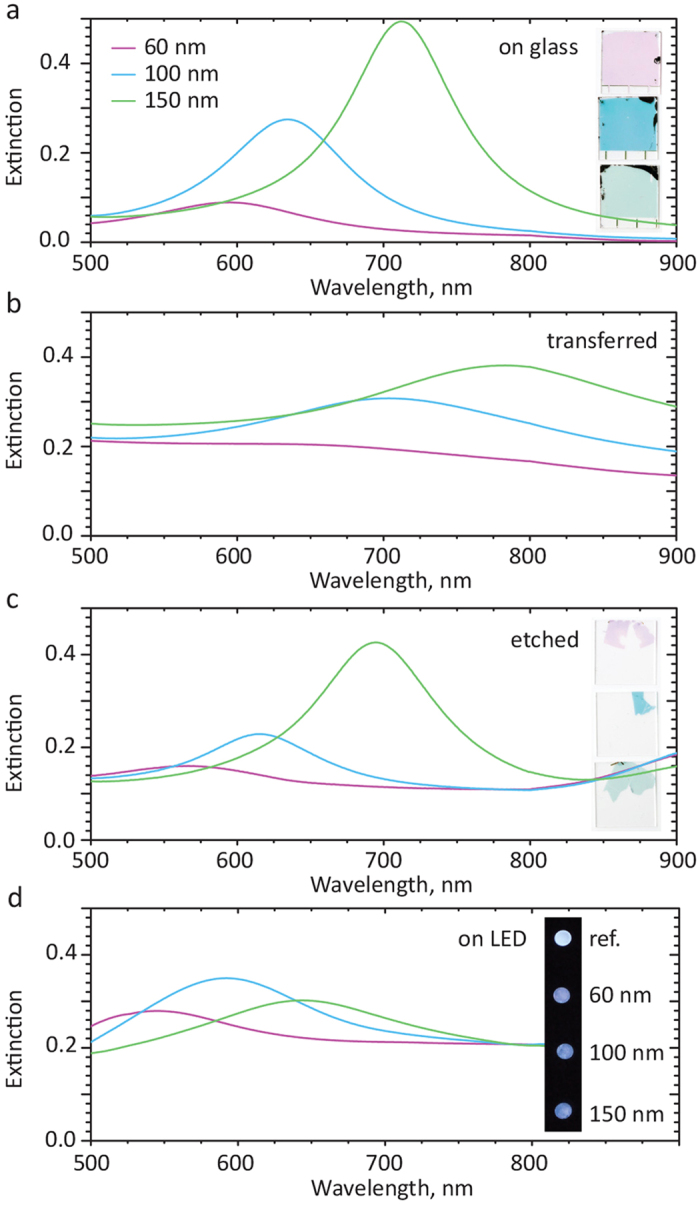
Following the pattern transfer with nanoplasmon spectroscopy and colorimetry. Optical absorbance of Au nanodisk antennas (**a**) on the glass substrate (inset - photograph of the corresponding samples); (**b**) on the glass substrate with SiO_2_ lift-off layer and e-beam evaporated carbon film; (**c**) after transfer to a host glass substrate and etch of the carbon layer (inset - photographs of the areas of the transferred patterns); (**d**) same nanoantennas, transferred onto a white LED (inset - the resulting color hues of the lit white LEDs).
