# Supplementary material for: Multiscale conformal pattern transfer
Source: Sci Rep. 2016 Jun 22;6:28490. doi: 10.1038/srep28490 (PMC4916408; doi:10.1038/srep28490)
Supplement: Supplementary Information [file srep28490-s1.pdf]

Supporting Information - Multiscale conformal pattern transfer

Kristof Lodewijks,[1,*] Vladimir Miljkovic,[1] Inès Massiot,[1] Addis Mekonnen,[1] Ruggero Verre,[1] Eva Olsson,[1] and Alexandre Dmitriev[1,†]

[1] *Department of Applied Physics, Chalmers University of Technology, 41296 Gothenburg, Sweden*

I. TRANSFER PROCESS STEPS

In figure S1 we illustrate the transfer protocol in practice for the transfer of 100 nm gold nanodisks onto a 100 Swedish kronor bank note (see figure 2c). The steps shown here are the steps as illustrated in figure 1, while the sample structure we start with was fabricated through HCL [1], on top of a 100/10 nm SiO_2/C stack, which was annealed in argon atmosphere before fabricating the nanoparticles.

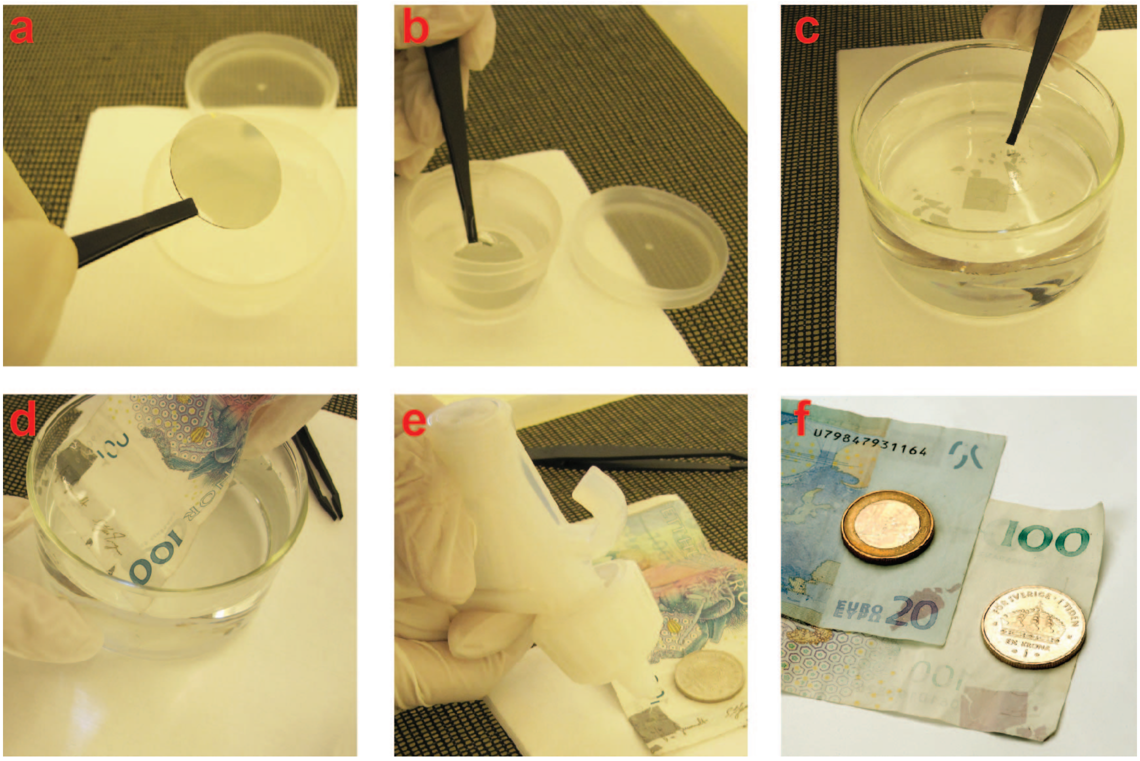

FIG. S1: Illustration of the transfer steps for HCL based disks onto a bank note. (a) The parent substrate is submerged into BOE etching solution to release the carbon film covered with gold particles. (b) The parent substrate is used to pick up the released carbon film with the lithographic pattern. (c) Upon immersion in DI water the carbon film floats on the water surface. (d) The bank note that serves as host substrate is used to pick up the pattern. (e) The note is dried under a mild nitrogen flow. (f) The bank note after oxygen plasma removal of the carbon film.

The subsequent processing steps are illustrated in figure S1:

a The sample is put into buffered oxide etch (BOE) solution, after patterning patches by means of a scalpel. The central patch is about 1 x 1 cm^2 in size.

---

*Corresponding author: lode@kristoflodewijks.be; Website: http://www.kristoflodewijks.be
†Corresponding author: alexd@chalmers.se

b The parent substrate is used to pick up the carbon film with the nanoparticle pattern. We can observe how the carbon film folds along one of the cuts made in the patterning stage, close to the tweezers.

c The different flakes are transferred onto the air/water interface, where we can see that the film breaks up in several pieces. The central piece to be transferred remained almost entirely intact and floats in the middle.

d The bank note is used to pick up the largest carbon flake.

e The transferred carbon film with the pattern is dried under a gentle nitrogen flow.

f The final structure is obtained after removing the carbon film by oxygen plasma etching, both for a 20 euro and a 100 Swedish kronor bank notes.

II. TRANSFER EXAMPLES

In this section we give an overview of some additional transfer examples to illustrate the power of the technique. Figure 2 illustrates how the optical properties are maintained for HCL based gold nanodisks of different sizes.

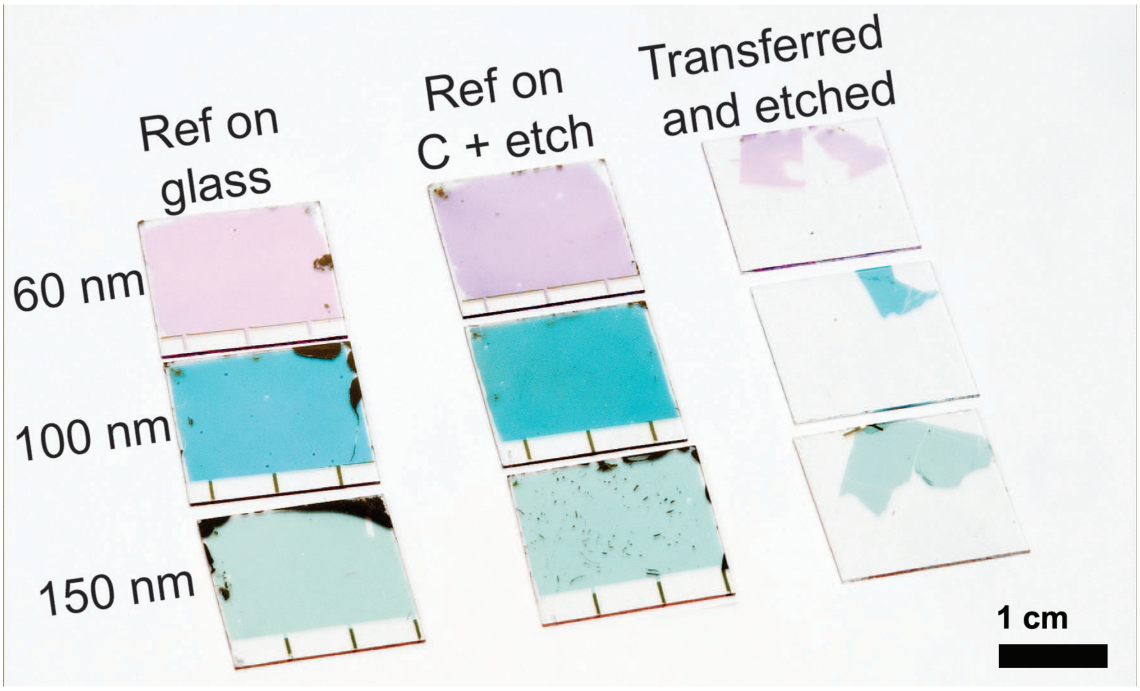

FIG. S2: Different sizes of HCL based gold nanodisks for comparison. From left to right columns: disks as fabricated on glass, disks as fabricated on glass and carbon film after etching the carbon film with oxygen plasma and disks after transfer and removal of the carbon film.

The samples shown in the left and right column of figure S2 are the samples used for optical characterization in figure 3, with the former being the reference structures on glass and the latter the samples after transfer and etching of the carbon film. The middle column shows gold nanodisks as fabricated on a carbon film after removal of the carbon layer by oxygen plasma etching. A slight color change can be observed for these samples, resulting in a small blue shift of the LSPR wavelength due to the fact that the surface of the gold is also oxidized. A similar trend is observed after the transfer, as can be seen in figure 3c. Note that the small dimensions of the patches are the result of pre-cutting patches with a scalpel, which can be seen clearly in the straight edges of the patches, and most clearly in the case of 150 nm disks, where the cutting lines are visible, but the flakes were not separated from each other during the transfer to the air/water interface.

In figure S3 we show microfluidic channels similar to those in figure 2c, coated with different sizes of HCL-based gold nanodisks. The arrows indicate different regions of interest on the samples: network of micro pillars that serve as an inlet filter (black), microfluidic channel with nanoparticle coated sidewalls (red) and patterning line where the different carbon patches were not separated (blue).

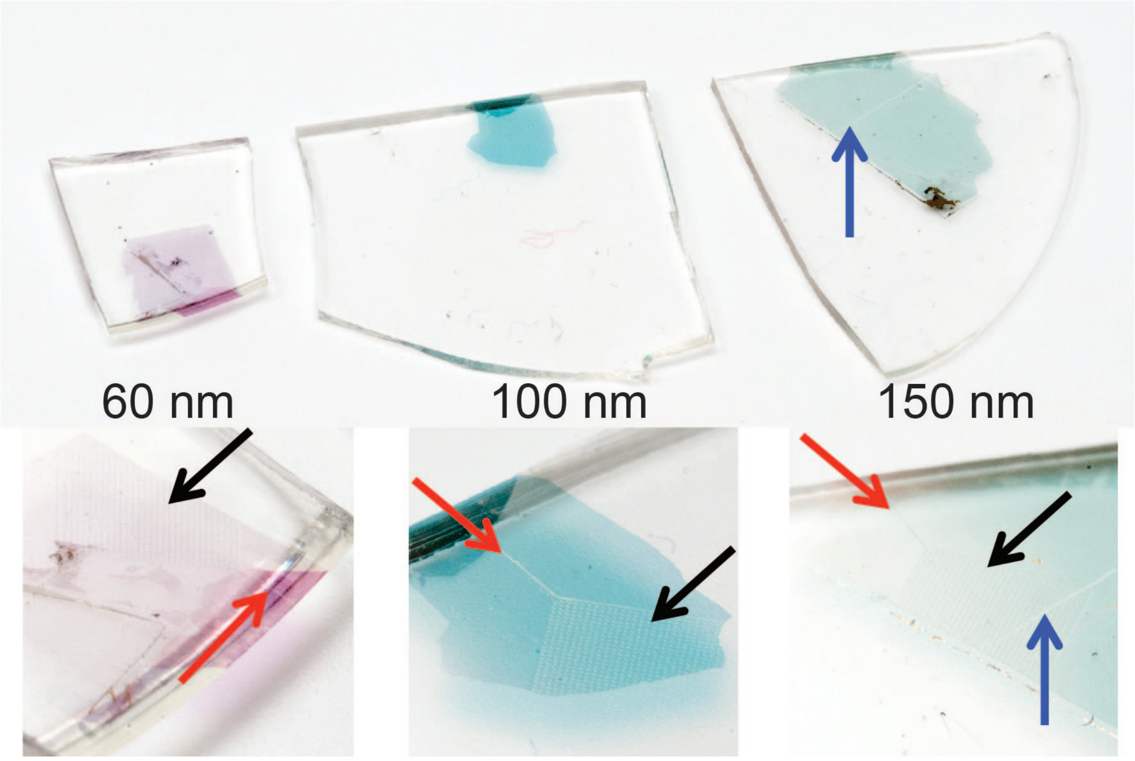

FIG. S3: Different sizes of HCL based gold nanodisks transferred onto microfluidic channels.

Figure S4 shows different examples for the transfer of a plasmonic nano hole film in platinum, with a hole diameter of 170 nm. The different panels show the following structures:

a SEM picture of the hole film transferred onto a different glass substrate after etching the carbon film with oxygen plasma.

b SEM picture of the pristine host substrate, illustrating the large surface roughness of polycrystalline silicon (poly-Si).

c Large scale SEM picture of the successful transfer onto rough poly-Si, illustrating how the structure nicely conforms to the intrinsic surface roughness.

d High magnification SEM picture zooming in onto an area where the pattern cracked due to the large roughness of the poly-Si.

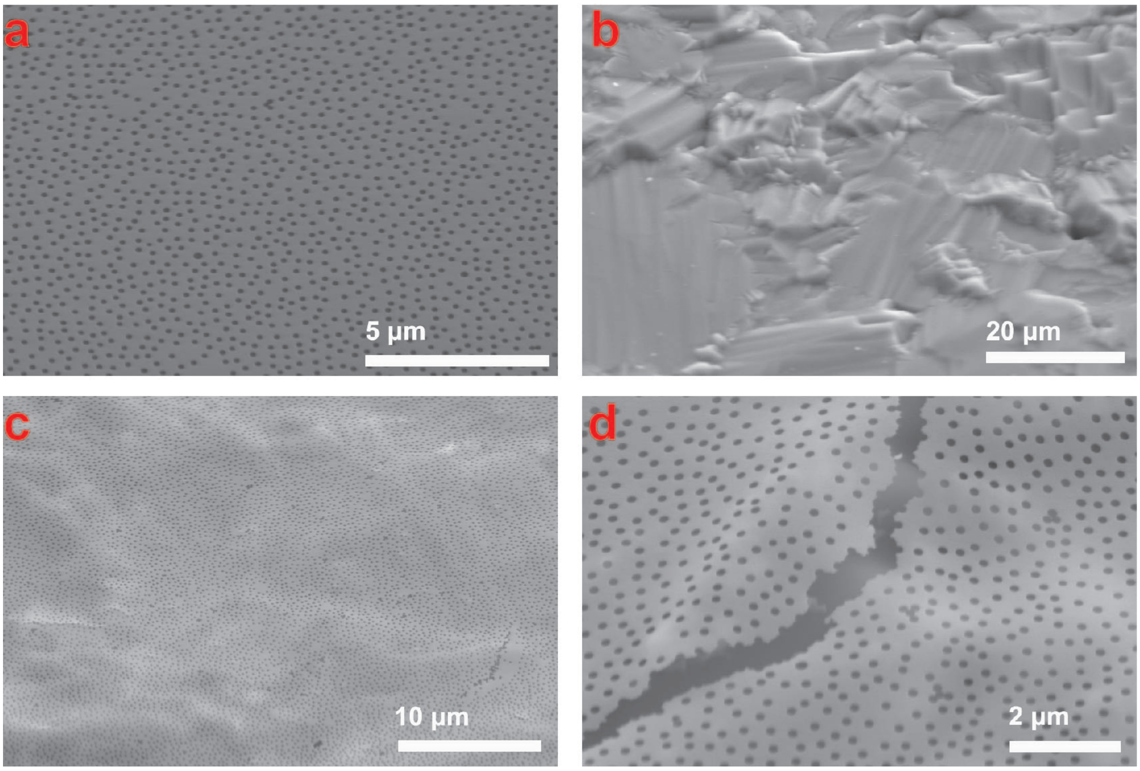

FIG. S4: SEM pictures of perforated platinum film transferred onto rough polycrystalline silicon substrates. (a) Reference pattern transferred onto a glass substrate. (b) Bare polycrystalline silicon reference sample. (c) Large area picture of transferred film without cracking. (d) Example of film cracking due to large host substrate roughness.

In figure S5 we study the LEDs coated with the Chalmers University of Technology logo (figure 2g), which were used in the projection setup and microscopy imaging. Panels a and b show the logo as fabricated on a glass substrate and after transfer to an LED respectively. In panel b, the natural roughness of the LED surface is clearly visible on the background, but nevertheless, the logo can be successfully transferred. Panel c illustrates how we can image the LED surface in a conventional microscope while using the illumination of the LED itself (20X magnification). In panel d we look at the same LED with low magnification, allowing us to visualise an array of 5 x 3 logos, all separated by 500μm. We can see how only the logo located on the top of the LED is in focus, while the other logo's are out of focus due to the large curvature of the LED surface. Here the roughness of the host substrate becomes even more apparent.

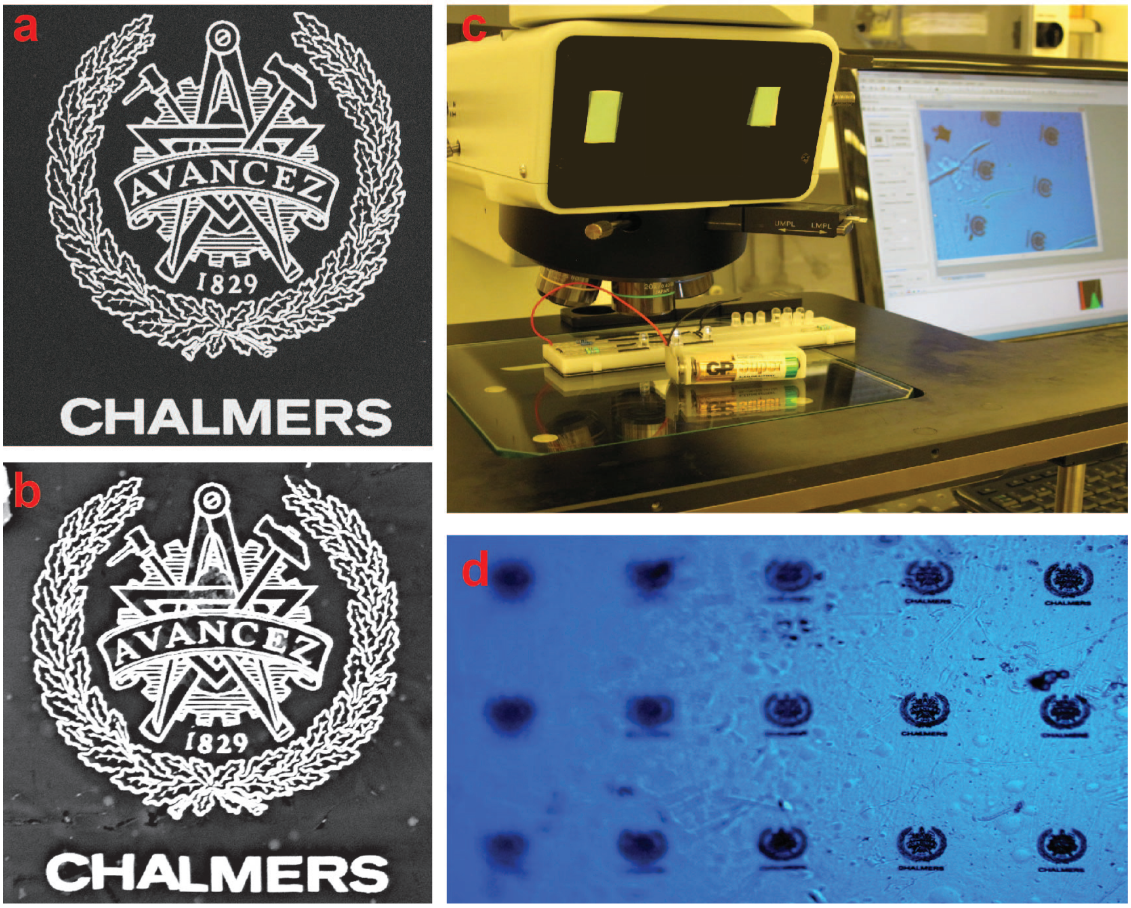

FIG. S5: Transfer of EBL based Chalmers logo onto LEDs. (a) SEM picture of logo's as fabricated on glass and carbon. (b) SEM picture of logo after transfer onto plastic LED, clearly showing the large roughness of the host substrate. (c) Illustration of the logo's imaged in a conventional microscope using the LED illumination. (d) Low magnification view of the array where only one logo is perfectly in focus. The large curvature of the host substrate causes defocussing of the logo's further away from the top of the LED.

In figure S6 we look in more detail to the transfer of HCL based gold nanodisks onto different commercial grade LEDs. The top from panel a shows the different fabrication steps on glass LEDs: an uncoated reference (left), a LED covered with a carbon film and 60 nm disks (middle) and a LED covered with 60nm gold disks after carbon film removal (right). Panels b and c show the LEDs coated with different sizes of nanodisks (figure 3d), without and with illumination respectively. The different colors can easily be observed in both pictures and in the spectral measurements. In panel d we show the edge of a carbon flake with 150 nm gold disks on top of the LED. On the left hand side we can see the natural roughness of the host substrate, while on the right hand side we can see that the particle film conforms very nicely to the surface. Panel e shows the same LED after removal of the carbon film, illustrating that despite the large roughness, the particle film covers the total surface of the LED.

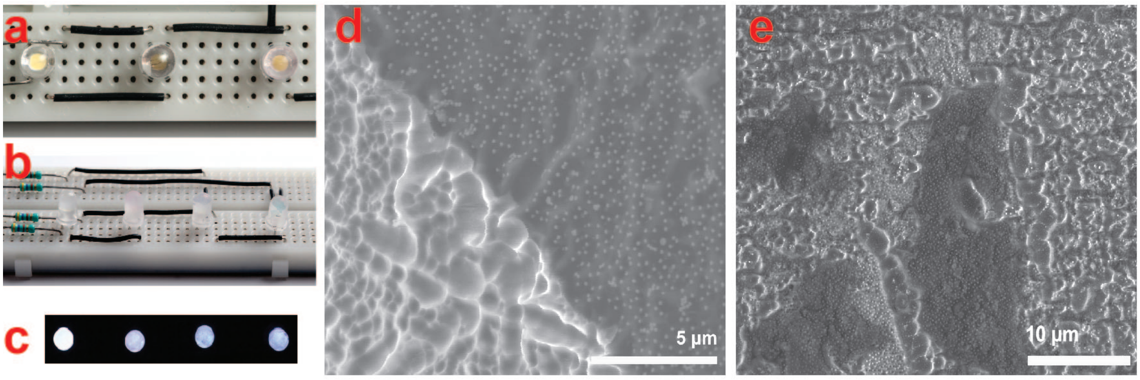

FIG. S6: Example of the transfer of HCL-based gold nanodisks onto LEDs. (a) Illustration of the different fabrication steps in glass LEDs: blank reference (left), carbon film with 60 nm disks after transfer (middle) and 60 nm disks after carbon film removal (right). (b) Different sizes of nanodisks after transfer to plastic LEDs, from left to right: blank reference LED, 60 nm particles, 100 nm particles and 150 nm particles after transfer and etch. (c) Top view picture of the same set of LEDs upon illumination. (d) SEM picture of the edge of a carbon flake with 150 nm nanodisks after transfer onto a plastic LED. The roughness of the LED surface can be clearly seen on the left side. (e) SEM picture of the same LED after carbon film removal.

[1] Fredriksson, H. et al., Hole-Mask Colloidal Lithography. *Adv. Mat.* **19**, 4297-4302 (2007)
